# Enveloped and non-enveloped virus survival on microfiber towels

**DOI:** 10.7717/peerj.15202

**Published:** 2023-04-13

**Authors:** Claire E. Anderson, Marlene K. Wolfe, Alexandria B. Boehm

**Affiliations:** 1 Stanford University, Stanford, CA, United States of America; 2 Emory University, Atlanta, GA, United States of America

**Keywords:** Virus, Fomites, Towels, phi6, ms2, Bacteriophage

## Abstract

**Background:**

Handwashing is an important intervention which can reduce indirect disease transmission, however soap and water for handwashing purposes is not available in some low-resource regions. When handwashing with soap and water is not possible, individuals may use alternatives such as the Supertowel (a microfiber towel with an antimicrobial coating). Testing of viral inactivation as a result of antimicrobial treatment on the Supertowel, however, has been limited. The goal of this study is to provide information about the performance of the Supertowel’s antimicrobial treatment against viruses, which will help inform the use of the towels as handwashing alternatives.

**Methods:**

We seeded the Supertowel and a regular microfiber towel with two bacteriophages (enveloped Phi6 and non-enveloped MS2) and monitored viral inactivation over time. Additionally, we assessed if temperature, humidity, whether the towel was initially wet or dry, or virus type had an effect on viral decay rate constants. Virus concentrations were measured repeatedly over 24 h.

**Results:**

We found that neither towel type (whether the towel was a Supertowel or a regular microfiber towel) nor humidity were significant variables in our model of decay rate constants (*P* = 0.06 and *P* = 0.22, respectively). We found that the variables of temperature, whether towels were initially wet versus dry, and virus type were significantly different from 0, suggesting that these variables explained variance in the decay rate constant (*P* = 6.55 × 10^−13^, *P* = 0.001, and *P* < 2 × 10^−16^, respectively). Higher temperatures, dry towels, and enveloped viruses all resulted in increases in the decay rate constant.

**Conclusions:**

Viruses seeded onto a Supertowel decay similar to viruses seeded onto a regular towel indicating that the virucidal potential of the Supertowel is minimal.

## Introduction

Indirect transmission of viruses occurs when viruses are released into the environment from an infected host, a susceptible individual comes into contact with the virus, and the susceptible individual becomes infected. Indirect transmission can occur through exposure to virus-laden air, food, water, or fomites (CDC, 2020). Hands can touch virus-contaminated fomites and viruses can be subsequently transferred to the skin (Anderson & Boehm, 2021). If the hand is then placed in the mouth or nose, for example, an individual may be exposed to the virus and may become infected. The hand may also transfer the virus to another surface. Given the potential for contaminated hands to contribute to indirect transmission of infectious disease, handwashing is promoted as an essential component of disease prevention.

Handwashing with soap and water is recommended by the World Health Organization (WHO) and Center for Disease Control and Prevention (CDC) (WHO, 2009; CDC, 2021), however, in low resource areas individuals may lack access to soap and water for hand hygiene (Freeman et al., 2014; Brauer et al., 2020; Yeasmin et al., 2021) so hand hygiene alternatives are needed. One such hand hygiene alternative is the Supertowel. The Supertowel is a reusable microfiber towel, treated with carbon chains affixed to positively charged nitrogen atoms, which are bonded to a silica layer in the microfibers of the towel (RealRelief, 2021; Torondel et al., 2021). The manufacturer indicates the positively charged layer is what interacts with negatively charged pathogens and creates the permanently bonded antimicrobial layer (RealRelief, 2021; Torondel et al., 2021). Manufacturer instructions indicate that the Supertowel should be dampened with water, wrung out, and then wiped over hands thoroughly for use as a substitute to handwashing with soap and water (RealRelief, 2021). Evidence suggests that the antimicrobial treatment on the Supertowel can inactivate bacteria (RealRelief, 2021; Torondel et al., 2021), however the ability of the Supertowel to inactivate viruses is not fully understood. String et al. (2021) reported that SARS-CoV-2, murine hepatitis virus (MHV), bacteriophage Phi6, and bacteriophage MS2 seeded onto a Supertowel were not reduced after 15 min in laboratory experiments. Temperature and humidity were not reported in the String et al. (2021) study and comparison to a regular microfiber towel was not made.

The goal of the present study is to investigate the inactivation of viruses Phi6 (a surrogate enveloped virus) and MS2 (a surrogate non-enveloped virus) seeded onto a Supertowel and a regular microfiber towel; inactivation was assessed at multiple time points over 24 h at a number of realistic environmental conditions. In particular, we carried out the experiments at three different temperatures (4 °C as winter temperature, 21 °C as temperate temperature, and 37 °C as tropical/desert temperature), two humidities (33% and 75%), and wet and dry towels. The results of study will inform the use of the Supertowel and regular towels as handwashing alternatives.

## Materials and Methods

### Virus preparation

Phi6 (NBRC 105899) was propagated in its host *Pseudomonas syringae* (*P. syringae*, ATCC 21781). Full descriptions of the culture media for the virus and host is described in Anderson and Boehm (Anderson & Boehm, 2021). Briefly, to propagate *P. syringae*, 30 mL of nutrient broth was inoculated with 20 µL of *P. syringae* stock and incubated at 30 °C while shaking at 75 revolutions per minute (rpm) for 48 h. Phi6 stock was created by mixing the soft agar of lysed plaque assay plate with phosphate buffered saline (PBS; Fisher BioReagents, Pittsburgh, PA, USA). The virus-agar-PBS mixture was centrifuged and the supernatant was filtered through a 0.2 µm pore-size membrane, concentrated using an Amicon^®^ Ultra-15 centrifugal filter unit (EMD Millipore, Burlington, MA, USA), and stored at −80 °C in a 15% glycerol solution. Phi6 stock concentration was approximately 10^11^ plaque forming units (PFU)/mL. MS2 (DMS No. 13767) was propagated in its host *Escherichia coli* (*E. coli*, ATCC 700891) based on Anderson and Boehm and EPA Method 1602 (EPA, 2001; Anderson & Boehm, 2021). Briefly, 20 mL of tryptic soy broth (TSB, pH of 7.3 ± 0.2) with ampicillin sodium salt and streptomycin sulfate was inoculated with 20 µL * E. coli* stock and incubated (without shaking) at 37 °C until the logarithmic growth phase. MS2 virus stock was created by mixing the soft agar of lysed plaque assay plate with PBS. The mixture was centrifuged, the supernatant was filtered through a 0.2 µL pore-size membrane, and the filtrate was stored at −80 °C in a 15% glycerol solution without ultrafiltration, because the concentration without ultrafiltration was adequately high. MS2 concentration of the stock was approximately 10^10^ − 10^11^ PFU/mL.

### Experimental procedure

A total of 24 experimental treatments were investigated (3 temperatures × 2 humidities × 2 towel types × wet/dry). Towel coupons were destructively sampled at fixed times up to 24 h and virus was recovered from the towel and enumerated. Each experimental treatment was repeated three times, creating three trials (trial A, trial B, and trial C) that serve as biological replicates. A full list of temperatures, towel conditions, humidity, and time points is in Table 1.

**Table 1:** Full list of sampling time points, temperature, humidity, towel type, and towel moisture tested.

**Time points sampled**	**Temperature**	**Relative humidity**	**Towel type**	**Towel moisture**
0 min 1 h 4 h 24 h	4C	High	Supertowel	Wet
				Dry
			Regular Towel	Wet
				Dry
		Low	Supertowel	Wet
				Dry
			Regular Towel	Wet
				Dry
0 min 1 h 4 h 24 h	21C	High	Supertowel	Wet
				Dry
			Regular Towel	Wet
				Dry
0 min 15 min 30 min 1 h 4 h 12 h 24 h	21C	Low	Supertowel	Wet
				Dry
			Regular Towel	Wet
				Dry
0 min 1 h 4 h 24 h	37C	High	Supertowel	Wet
				Dry
			Regular Towel	Wet
				Dry
		Low	Supertowel	Wet
				Dry
			Regular Towel	Wet
				Dry

Dry and wet new, unused Supertowels (Real Relief, Kolding, Denmark) and new, unused regular towels (Real Relief, Kolding, Denmark) were used in the experiments. The only difference between the Supertowels and the regular towels was that the Supertowel had an antimicrobial coating per the manufacturer. Towels were cut into 2 cm^2^ coupons using sterile scissors. Dry towels were used as purchased. A subset of the coupons was wet using 500 µL of autoclaved deionized (DI) water at the start of experiments (towels were not re-wet throughout the experiment). Thereafter, 100 µL of virus suspension (50 µL of approximately 10^10^ PFU/mL Phi6 stock and 50 µL of approximately 10^9^ PFU/mL MS2 stock) was applied to the coupons. The towel was stored in an airtight tupperware containers (Rubbermaid) at the specified humidity (high (75% relative humidity (RH)) or low (33% RH)) and temperature (4 °C, 21 °C, or 37 °C) for up to 24 h. RH was maintained using saturated salt solutions of MgCl_2_ for 33% RH and NaCl for 75% RH within the containers (Greenspan, 1977). Temperature and humidity were monitored using a ThermoPro TP49 digital hygrometer placed in the containers.

Virus was recovered from towels at each time point by submerging the entire towel coupon in 4 mL of tryptic soy broth (TSB) and vortexing for 20 s. The coupon was then carefully removed from the TSB and inserted into a 5 mL syringe and excess liquid was pressed out of the coupon and into the TSB (see Fig. S1). This allowed us to recover nearly 4 mL of TSB sample. The TSB samples were kept at 4 °C and plated in a double layer plaque assay appropriate for each virus type within 6 h of sample collection.

Negative controls were included with each experiment and consisted of a Supertowel coupon, stored in environmental conditions for 24 h, and put through the viral recovery process before sample plating. Positive controls were included with each experiment and consisted of plating diluted −80° C viral stock, which we expected to have a concentration of ∼10^11^ PFU/mL.

### Virus plaque assay quantification

Viral concentration was evaluated using double-layer plaque assays to quantify infective viruses using 60 mm diameter Petri dishes. To enumerate Phi6, 100 µL of *P. syringae* host and 100 µL of diluted sample were added to soft nutrient agar (0.3% agar), then poured onto hard nutrient agar plates (2.3% agar). Phi6 plates were incubated upright overnight at 30 °C, then PFUs were counted. To enumerate MS2, 200 µL of log-phase *E. coli* and 300 µL of diluted sample were added to soft tryptic soy agar (0.7% agar), then poured onto hard tryptic soy agar plates (1.5% agar). MS2 plates were incubated upside-down overnight at 37 °C, then PFUs were counted.

The TSB samples were diluted prior to plating according to preliminary experiments which provided information on how to dilute the virus-containing TSB at each time point so as to obtain a countable number (between 0 and 500) of PFUs. Dilutions ranged from undiluted to 1:10^6^ for MS2 and 1:10^7^ for Phi6. Although 500 PFU is a large number of plaques to count, plaques formed by these viruses with the hosts are small, and 500 PFU was deemed countable by the technicians. Plates with more than 500 PFUs were classified as TNTC (too numerous to count). In some instances, all the dilutions we plated were TNTC and in these cases, the plaque assay was repeated using the archived sample which was held at 4 °C using greater dilutions. Repeated plaque assays were completed within 24 h of sample collection. Three to four dilutions were plated for each sample.

### Data analysis

Figures and statistical tests were performed with R (R Core Team, 2020). A Wilcoxon rank sum test was used as a non-parametric alternative to a *t*-test to determine if the median viral recovery differed between Supertowel and regular towels.

#### First order inactivation rate constant (k)

To calculate *k*, we used the first order inactivation model described in Eq. (1).

(1) \begin{eqnarray*}ln \left( \frac{{N}_{t}}{{N}_{0}} \right) =-kt\end{eqnarray*}

where *N*_0_ is the average initial viral concentration (PFU/mL) of replicates recovered from the towel sample at *t* = 0, *N*_*t*_ is the average viral concentration (PFU/mL) of replicates recovered from the towel at each time point, *t* is time (hour), and *k* is the first-order rate constant (h^−1^). Using the left-hand-side of Eq. (1) as our dependent variable and* t* as our independent variable, we were able to determine the slope of the model (*-k*) using a linear regression fit function in R.

#### Multiple regression analysis

We investigated the following independent variables which may affect the dependent variable *k:* temperature, humidity, towel type, wet versus dry towels, and virus type. The latter four variables were categorical and temperature was continuous. The relationship between the first-order rate constant *k* and temperature is typically described through the Arrhenius Eq. (2), where *A* is the pre-exponential factor, *E*_*a*_ is the activation energy (J/mol), *R* is the universal gas constant (J/(molK)), and *T* is the absolute temperature (K).

(2) \begin{eqnarray*}k=A{e}^{-{E}_{a}/RT}.\end{eqnarray*}

The Arrhenius equation in the form of a linear equation is shown in Eq. (3):

(3) \begin{eqnarray*}ln(k)=ln(A)- \frac{{E}_{a}}{RT} .\end{eqnarray*}

To evaluate the effect of the categorical variables, we assigned a dummy variable to the variable. These dummy variables were used in addition to the Arrhenius equation to develop Eq. (4). In this equation, ln(*A*) is replaced with *a*, and −*E*_*a*_/*R* is replaced with *β*_*T*_.

(4) \begin{eqnarray*}ln(k)=\alpha +({\beta }_{T}\ast \frac{1}{T} )+({\beta }_{RH}\ast {X}_{RH})+({\beta }_{TT}\ast {X}_{TT})+({\beta }_{Dry}\ast {X}_{Dry})+({\beta }_{Virus}\ast {X}_{Virus}).\end{eqnarray*}

Multiple linear regression assumes that the relationships between the dependent variables and ln(*k*), our independent variable, are linear, that observations are independent, and that independent variables are not highly correlated with one another. The linear regression also assumes that model residuals are normally distributed and that the variance of error is similar across independent variables. Confirmation of these assumptions is detailed in the SI.

We conducted a power analysis to determine the sensitivity of our results (details in SI). Using an alpha of 0.05 for specificity, a power of 0.80, and our 5 predictors, our total sample size of 48 data points was able to measure an effect size (Cohen’s *f*^2^) of 0.30. Cohen’s *f*^2^ is a unitless value, calculated with model R^2^ values (see SI for complete calculation) (Selya et al., 2012). Cohen’s *f*^2^ value of 0.02–0.15 is typically a small effect, 0.15–0.35 is a medium effect, and >0.35 is a large effect (Cohen, 1988; Selya et al., 2012). Therefore, our study is powered to identify medium effects.

#### Data preparation

In instances where there were zero plaques in all plates of the undiluted samples, the sample was recorded as a non-detect and 0.5 (1/2 the limit of detection, 1 PFU) was substituted as the undiluted sample PFU. Outliers of *k*, determined using a threshold of four times the mean Cook’s Distance, were removed from the data set of *k* values.

## Results

All negative controls for the experiments had zero plaques and all stock concentrations results were as expected (10^11^ PFU/mL for Phi6 and MS2 stock) indicating that the assays performed as expected with no contamination. In total, we measured Phi6 and MS2 each in 324 samples. Samples were below the limit of detection and substituted with 0.5 PFU 40 times out of 324 data points for Phi6 and 0 times for MS2. Median virus recovery using the recovery procedure was 0.69% for Supertowels (*n* = 72) and 0.72% for regular towels (*n* = 72) (raw data for recovery calculations are shown in Fig. S2). A Wilcoxon rank sum test between the two groups shows that the median viral recovery between the Supertowels and regular towels is not significantly different (*P* = 0.91).

**Figure 1:**
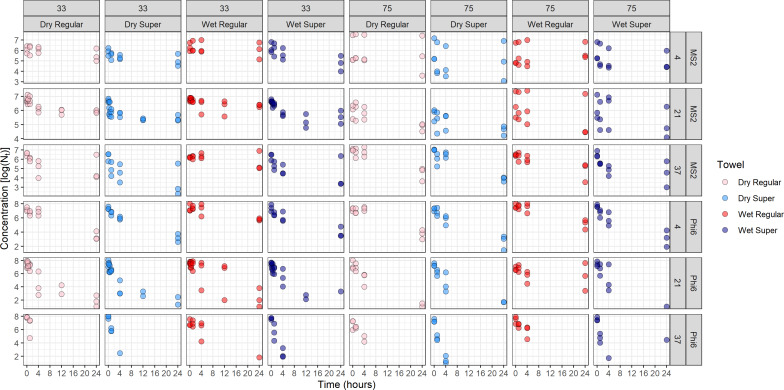
Virus concentrations over the course of the experiments. Results are grouped by virus, then subdivided into towel type, temperature, and relative humidity. Pink results are for dry regular towels, light blue are for dry Supertowels, red are for wet regular towels, and dark blue are for wet Supertowels.

For all experiments, virus concentrations tended to decrease with time (Fig. 1). 38 of 48 *k* values were statistically different from 0 (*P* < 0.05). Of the 38 *k* values that were significantly different from 0, 37 were positive indicating decreases in concentration with time while 1 was negative indicating an increase over time. Across all tested conditions where *k* was significantly different from 0, *k* varied from −0.04 to 0.27h^−1^ for MS2 and 0.16 to 3.19 h^−1^ for Phi6 (Table 2).

**Table 2:** Plaque assay *k*, *p*-value, and standard error results for each treatment type.

**Treatment**	**MS2**			**Phi6**		
	k **(h**^−1^**)**	*P*-value	Standard Error	**k** (h^−1^**)**	*P*-value	**Standard Error**
Dry Regular, 4C, 33%RH	0.06	1.67 × 10^−3^	0.01	0.35	6.54 × 10^−7^	0.03
Dry Super, 4C, 33%RH	0.06	2.92 × 10^−2^	0.02	0.37	3.81 × 10^−7^	0.03
Wet Regular, 4C, 33%RH	0.03	2.47 × 10^−1^	0.02	0.16	7.51 × 10^−5^	0.02
Wet Super, 4C, 33%RH	0.13	1.30 × 10^−4^	0.02	0.29	6.44 × 10^−6^	0.03
Dry Regular, 4C, 75%RH	0.05	1.57 × 10^−1^	0.03	0.34	1.33 × 10^−6^	0.03
Dry Super, 4C, 75%RH	0.03	5.83 × 10^−1^	0.05	0.42	1.01 × 10^−5^	0.05
Wet Regular, 4C, 75%RH	-0.04	3.31 × 10^−2^	0.02	0.24	2.74 × 10^−6^	0.03
Wet Super, 4C, 75%RH	0.05	3.36 × 10^−2^	0.02	0.38	4.37 × 10^−5^	0.06
Dry Regular, 21C, 33%RH	0.09	6.36 × 10^−5^	0.02	0.54	3.15 × 10^−6^	0.08
Dry Super, 21C, 33%RH	0.09	1.16 × 10^−3^	0.02	0.56	4.48 × 10^−6^	0.08
Wet Regular, 21C, 33%RH	0.05	3.48 × 10^−2^	0.02	0.49	6.33 × 10^−6^	0.08
Wet Super, 21C, 33%RH	0.12	1.58 × 10^−4^	0.02	0.54	1.64 × 10^−5^	0.09
Dry Regular, 21C, 75%RH	0.11	9.16 × 10^−7^	0.01	0.58	5.20 × 10^−6^	0.06
Dry Super, 21C, 75%RH	0.09	5.59 × 10^−3^	0.02	0.49	5.10 × 10^−4^	0.09
Wet Regular, 21C, 75%RH	0.09	8.42 × 10^−3^	0.03	0.10	2.66 × 10^−1^	0.08
Wet Super, 21C, 75%RH	0.09	4.17 × 10^−2^	0.04	0.57	1.29 × 10^−3^	0.12
Dry Regular, 37C, 33%RH	0.11	1.32 × 10^−1^	0.07	3.12	1.78 × 10^−1^	1.91
Dry Super, 37C, 33%RH	0.19	7.39 × 10^−2^	0.10	2.98	1.09 × 10^−4^	0.27
Wet Regular, 37C, 33%RH	0.06	1.18 × 10^−1^	0.03	0.56	2.23 × 10^−4^	0.09
Wet Super, 37C, 33%RH	0.13	1.49 × 10^−1^	0.08	2.89	1.99 × 10^−4^	0.41
Dry Regular, 37C, 75%RH	0.24	1.26 × 10^−5^	0.03	1.55	1.56 × 10^−3^	0.28
Dry Super, 37C, 75%RH	0.27	2.44 × 10^−5^	0.04	3.19	2.97 × 10^−5^	0.34
Wet Regular, 37C, 75%RH	0.16	1.30 × 10^−3^	0.04	0.94	6.18 × 10^−4^	0.16
Wet Super, 37C, 75%RH	0.14	4.38 × 10^−2^	0.06	0.22	3.60 × 10^−1^	0.22

Four outliers were removed from the *k* data set. We applied a multiple linear regression model to test whether different experimental factors including type of towel, wet versus dry, temperature, RH, and virus type were associated with *k*. Full results of the multiple linear regression model, including the variable estimates, standard errors, and *p*-values, are shown in Table 3. The adjusted R^2^ value of the model is 0.91. The *F*-statistic is 90.6 on 37 degrees of freedom.

**Table 3:** Multiple linear regression results. Shown are the variables of interest, the terms they represent from Eq. (4), their estimate, standard error, and *p*-value. The unit on the estimate and the standard error is the unit of the LHS of Eq. (4) except in the case of temperature where the unit of the estimate and standard error are the unit of the LHS/Kelvin.

**Variable**	**Term**	**Estimate**	**Standard error**	*P*-value
Intercept	α	10.2	1.21	3.54 × 10^−10^
Temperature	*β*_T_	-3.78 × 10^3^	352	6.55 × 10^−13^
Humidity	*β*_RH_	0.14	0.11	0.22
Towel type	*β*_TT_	-0.21	0.11	0.059
Wet or dry towel	*β*_Dry_	0.40	0.11	0.001
Virus type	*β*_V irus_	2.07	0.11	<2 × 10^−16^

We found that coefficients for temperature, for the dummy variable describing whether towels were initially wet versus dry, and for the dummy variable describing virus type were significantly different from 0, suggesting that these variables explained variance in ln(*k*) (*P* = 6.55 × 10^−13^, *P* = 0.001, and *P* < 2 × 10^−16^, respectively). Towel type was not a significant variable in the model nor was RH (*P* = 0.06 and *P* = 0.22, respectively). The unitless measure of effect size, Cohen’s* f*^2^, of temperature, wet versus dry towels, and virus type were 3.00 (large effect), 0.33 (medium effect), and 9.46 (large effect), respectively. The results of the analysis indicate that *k* values tended to be larger at higher temperatures, on dry towels, and for Phi6 compared to MS2.

## Discussion

Decay rate constants (*k*) of an enveloped and non-enveloped bacteriophage seeded on Supertowels were not different from those seeded onto a regular towel without the antimicrobial coating. This suggests that the antimicrobial coating on the Supertowel does not have enhanced antiviral activity relative to a regular towel. A previous study (String et al., 2021), investigated the Supertowel efficacy as an antimicrobial surface and found that the Supertowel resulted in a log reduction of −0.17 for infectious SARS-CoV-2, 0.08 for MHV, 0.98 for Phi6, and −0.58 MS2 after 15 min of exposure to the Supertowel. Negative log reduction values observed for SARS-CoV-2 and MS2 indicate an increase in viral concentration after 15 min which might be due to variability in the measurement methods since the virus cannot multiply on the towel surface.

An effective antimicrobial surface, according to the United States Environmental Protection Agency’s (US EPA’s) interim guidelines for effective antimicrobial surface coatings, should result in a minimum 3 log reduction after 1–2 h (EPA, 2020). Supertowel results from our study show the median log reduction across conditions (*n* = 72) after one hour was 0.81 log, suggesting that the Supertowel would not be an effective antimicrobial surface according to US EPA standards.

When the Supertowel and a regular towel were actually used in practice for handwashing, Anderson et al. (2023) found that the Supertowel and regular microfiber towels performed similarly in reducing viruses on hands of volunteers. The Supertowel manufacturer reports that the Supertowel is an effective antimicrobial surface for bacteria and fungi (RealRelief, 2021). A volunteer handwashing study by Torondel et al. (2021) also found the Supertowel was an effective handwashing alternative to the use of soap and water when tested against *Escherichia coli* seeded onto volunteer hands. Therefore, while the results from this study indicate that the Supertowel antimicrobial surface may not be effective against viruses, it could still be effective against bacteria and fungi.

The decay rate constant of MS2 and Phi6 on the microfiber towels was influenced by temperature, virus type, and whether the towel was initially wet or dry. As temperature increased, *k* also increased. This is consistent with previous studies which found that temperature and viral decay rate constants are positively associated for viruses on surfaces (Casanova et al., 2010; Chan et al., 2011; Prussin et al., 2018; Chin et al., 2020; Hessling, Hoenes & Lingenfelder, 2020; Morris et al., 2021). Increases in temperature can speed up biochemical reactions that might affect virus degradation, as demonstrated through use of the Arrhenius equation to describe viral decay rate constants (Prussin et al., 2018; Hessling, Hoenes & Lingenfelder, 2020; Morris et al., 2021). Additionally, we found that virus-type influenced the decay rate constant; enveloped virus decay rate constants were greater than non-enveloped viruses decay rate constants. The Cohen’s *f*^2^ effect size of the virus-type variable was 9.46 (unitless), which is considered a large effect size, and was the largest effect size of the variables we measured in this study. Previous studies have also found that enveloped virus inactivation is greater than non-enveloped virus inactivation on surfaces (Kramer, Schwebke & Kampf, 2006; Firquet et al., 2015). This might be explained by the increased sensitivity of the lipids in the viral envelope to environmental stressors in this study; additionally the two viruses have different genomes and protein capsids which also might have differential susceptibility to environmental stressors in this project. Finally, we found that viruses seeded on towels which were initially dry had greater decay rate constants than viruses seeded on towels which were initially wet. The relationship between viruses seeded onto initially wet or initially dry porous surfaces and viral decay rate constants has not been reported previously to our knowledge, although repeated drying cycles have been shown to reduce virus concentrations (Firquet et al., 2015). Desiccation is a known environmental stressor for many microorganisms (Vasickova et al., 2010).

One limitation of this study is the use of non-pathogenic bacteriophages as surrogates to human pathogenic viruses. Although MS2 and Phi6 have been previously used as model viral pathogens in surface inactivation studies investigating virus survival on surfaces (Casanova et al., 2010; Prussin et al., 2018; String et al., 2021), their behavior is not representative of all pathogenic viruses (Prussin et al., 2018), and further experiments should be conducted if a single virus is of concern. A second limitation is that this study used two RH points to analyze the influence of humidity on the decay rate constant. Although our study found that the decay rate constant of viruses on towels was not significantly influenced by humidity, previous studies found that the relationship between humidity and virus survival on surfaces was non-monotonic (U-shaped) (Casanova et al., 2010; Chan et al., 2011; Prussin et al., 2018; Morris et al., 2021). However, the influence of humidity on survival rate could also be related to virus species (Kramer, Schwebke & Kampf, 2006). An additional consideration when interpreting the results of this study is that we aimed to evaluate the efficacy of the Supertowel independent of actual use scenarios, where efficacy may be affected by friction or mechanical removal from wiping hands with the towel. Efficacy results from using the towel in practice are investigated in Anderson et al. (2023) and Torondel et al. (2021), as discussed previously.

## Conclusions

The goal of this study was to seed the Supertowel and a regular microfiber towel with two bacteriophages (Phi6 and MS2) and monitor viral inactivation over time, which will help inform the use of the towels as handwashing alternatives. Results suggest that the Supertowel provides inconsistent inactivation of viruses, as differences in viral decay rate constants were not explained by towel type. These viral results differ from tests with bacteria, which suggested the Supertowel was an effective antimicrobial tool (RealRelief, 2021; Torondel et al., 2021). Results of this study also add evidence that high temperatures result in larger decay rate constants, that enveloped viruses have higher decay rate constants than non-enveloped viruses, and that virus decay rate constants are larger on porous materials which are dry. Together, these results suggest that the decay rate constants of viruses on porous surfaces, like towels, will be greater for enveloped viruses in hot, dry conditions. Future researchers should monitor these conditions when investigating decay rate constants so they can appropriately frame their results.

##  Supplemental Information

10.7717/peerj.15202/supp-1 Supplemental Information 1 Supplemental Figures and Tables

## Additional Information and Declarations

### Data Availability

The following information was supplied regarding data availability: The data is available at the Stanford Digital Repository: Anderson, C., Tong, J., Zambrana, W., Boehm, A., and Wolfe, M. (2022). Data Set for: Enveloped and non-enveloped virus survival on microfiber towels. Stanford Digital Repository. https://purl.stanford.edu/gk906zk5623.
