# Supplementary material for: Enveloped and non-enveloped virus survival on microfiber towels
Source: PeerJ. 2023 Apr 13;11:e15202. doi: 10.7717/peerj.15202 (PMC10106082; doi:10.7717/peerj.15202)
Supplement: Supplemental Information 1 [file peerj-11-15202-s001.docx]

**Supplemental Information**

**Enveloped and non-enveloped virus survival on microfiber towels**

Claire E. Anderson^1^, Marlene K. Wolfe^1,2*^, Alexandria B. Boehm^1*^

^1^ Department of Civil and Environmental Engineering, Stanford University, Stanford, CA, 94305

^2^ Gangarosa Department of Environmental Health, Rollins School of Public Health, Emory University, Atlanta, GA, USA, 30322

*Authors to whom correspondence should be addressed:

Alexandria Boehm, [aboehm@stanford.edu](mailto:aboehm@stanford.edu), 650-724-9128;

Marlene Wolfe, [marlene.wolfe@emory.edu](mailto:marlene.wolfe@emory.edu), 617-583-2401

The supplemental material contains the following information, as referenced in the main article:

| Figure S1: Experimental Setup Photos……………….……………………………….….. | S2 |
| --- | --- |
| Table S1: Multiple Linear Regression Assumptions Check……………………………… | S3 |
| Power Analysis…………………………………………………………………………… | S4 |
| Table S2: *f ^2^* calculation variables…………………………...……………………. | S5 |
| Figure S2: Applied concentrations to time zero concentrations .……………………….... | S6 |
|  |  |
|  |  |

**Figure S1: Experimental Setup Photos**

| 1. **Application of the virus onto towel** | 1. **TSB + towel vortex** | 1. **TSB recovery from towel through syringe** |
| --- | --- | --- |
| **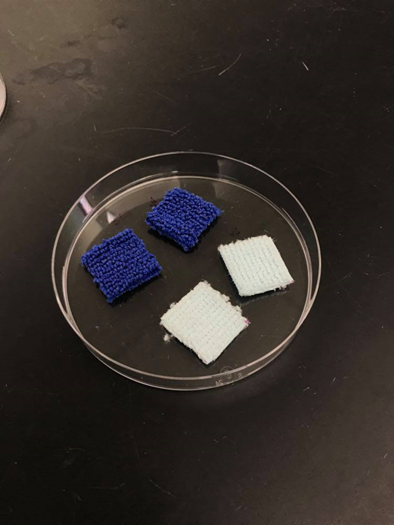** | **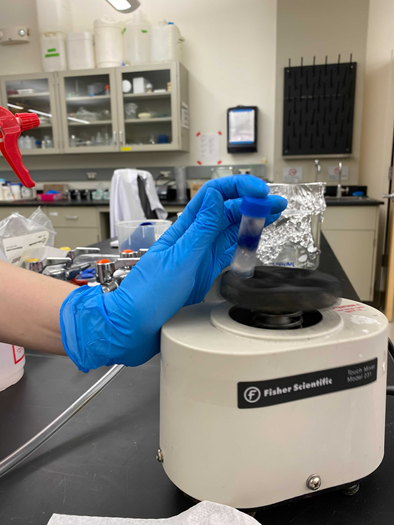** | **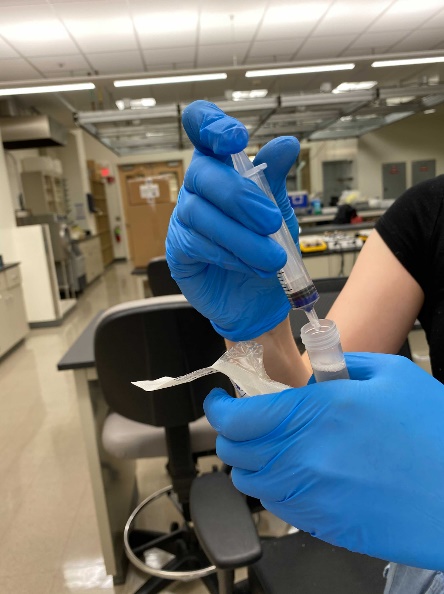** |

**Table S1: Multiple Linear Regression Assumptions Check**

| **Assumption** | **Description** | **Test** | **Result** |
| --- | --- | --- | --- |
| Linear Relationship | Linear relationship between each independent variable and the dependent variable | Dependent variable versus independent variable plot | Plots for each variable visually appear linear. |
| No Multicollinearity | Independent variables are not related to one another | Variance Inflation Factor (VIF) calculation | All VIF values are equal to 1, indicating no multicollinearity. |
| Homoscedasticity | Residual variance is constant across independent variables | Standardized residuals versus predicted values plot followed by Breusch-Pagan test | Breusch-Pagan test = 0.35 with all variables, indicating that homoscedasticity is present. |
| Multivariate Normality | Residuals are normally distributed | Q-Q plot followed by Shapiro-Wilk’s test | Shapiro-Wilk’s test: 0.17 with all variables, indicating that residuals are generally normally distributed. |

**Power Analysis**

Sensitivity power analysis for this study was calculated using G*Power (Statistical power analyses using G*Power 3.1: Tests for correlation and regression analyses, version 3.1.9.6; University of Kiel, Germany).

The following variables were input into the program:

| **Variable** | **Input** |
| --- | --- |
| Test family | F tests |
| Statistical test | Linear multiple regression: Fixed model, R^2^ deviation from zero |
| Type of power analysis | Sensitivity: Compute the required effect size - given alpha, power, and sample size |
| Alpha error probability | 0.05 |
| Power | 0.80 |
| Total sample size | 48 |
| Number of predictors | 5 |

The effect size (Cohen’s *f ^2^*) the sample population was able to detect was 0.30. Our study has the power to measure a large effect size, as outlined by the conventional values proposed by Cohen (1988), where effect sizes were defined as 0.02 as small, 0.15 as medium, and 0.35 as large.

The local effect size (Cohen’s *f ^2^*) from each variable was calculated using Equation S1. RAB2 is equal to the proportion of variance of the entire model (including the variable of interest *B* and all the set of all other variables *A)*. $R_{A}^{2}$ is equal to the proportion of variance of the entire model excluding the variable of interest *B* and only including the set of all other variables *A.*

| $f^{2}=\frac{R_{AB}^{2}-R_{A}^{2}}{1-R_{AB}^{2}}$ | Eq. S1 |
| --- | --- |

________________________

^1^ Selya, Arielle S., et al. “A Practical Guide to Calculating Cohen’s F2, a Measure of Local Effect Size, from Proc Mixed.” *Frontiers in Psychology*, vol. 3, 2012, https://doi.org/10.3389/fpsyg.2012.00111.

$R_{AB}^{2}$, $R_{A}^{2}$, and *f ^2^* values for each variable are shown in Table S3. The local effect size is the measure of variance accounted for by our variable of interest versus other variables. A high local effect size indicates that the covariate of interest has a large effect on the model.

**Table S2: *f ^2^* calculation variables**

| Variable | RAB2 | RA2 | f 2 |
| --- | --- | --- | --- |
| Temperature | 0.914 | 0.657 | 3.00 |
| Humidity | 0.914 | 0.913 | 0.015 |
| Towel type | 0.914 | 0.908 | 0.074 |
| Wet or dry towel | 0.914 | 0.886 | 0.33 |
| Virus | 0.914 | 0.104 | 9.46 |

**Figure S2: Applied concentrations and time zero concentrations**

Applied sample concentrations are plotted with an unfilled circle, while time zero concentrations are filled.

**
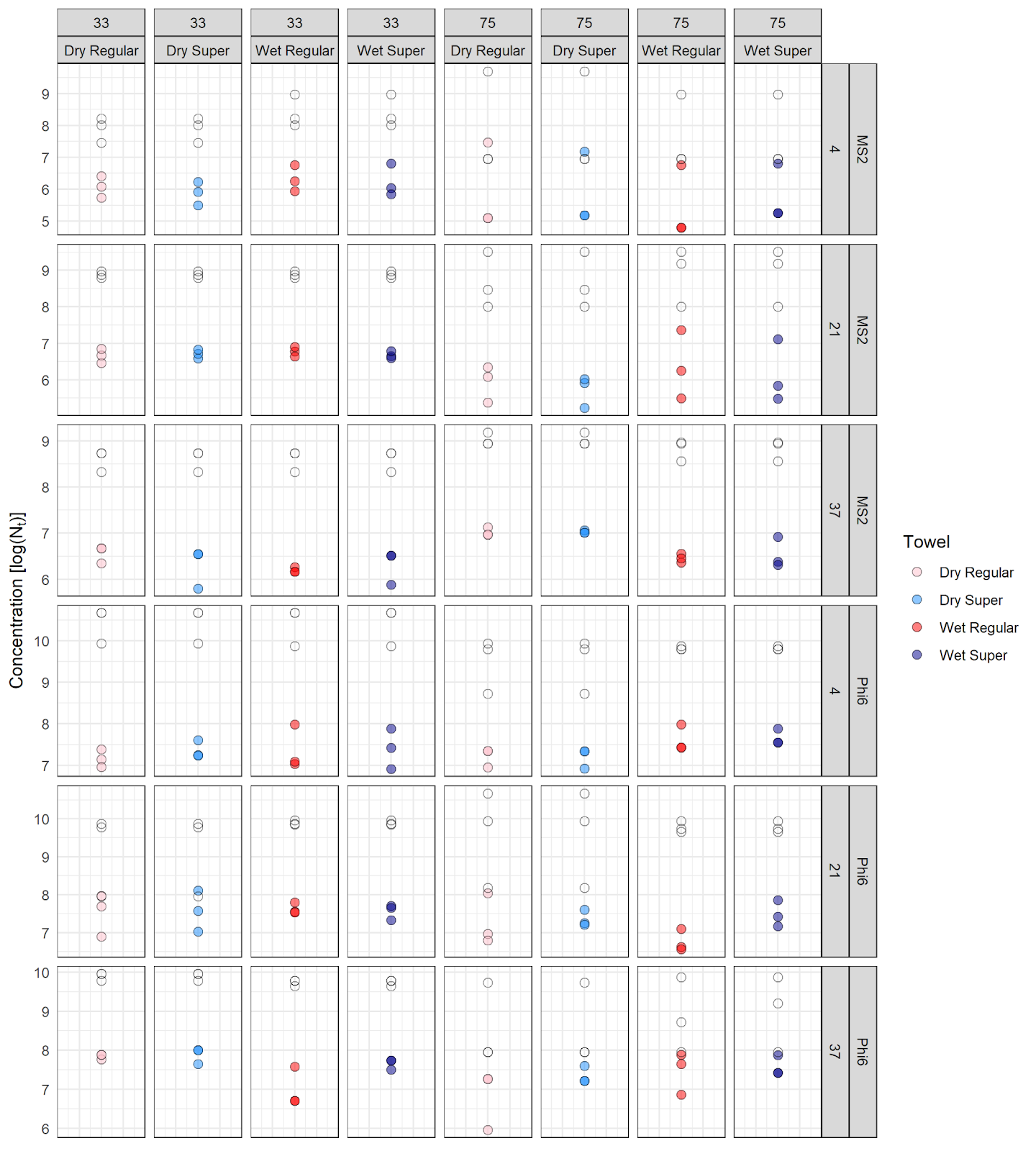
**
